# Food nanoparticles from rice vinegar: isolation, characterization, and antioxidant activities

**DOI:** 10.1038/s41538-021-00118-y

**Published:** 2022-01-11

**Authors:** Zhaoshuo Yu, Ying Tan, Sihao Luo, Jingru Zhou, Tianhao Xu, Jianqiao Zou, Lijing Ke, Ji Yu, Suyun Zhang, Jianwu Zhou, Pingfan Rao, Jiaxing Li

**Affiliations:** 1 SIBS-Zhejiang Gongshang University Joint Centre for Food and Nutrition Sciences, Zhejiang Gongshang University, Hangzhou, 310012 China; 2 Institute of Food Science, Jishou University, Jishou, 416000 Hunan China; 3 Hunan Salt Industry Co., Ltd., Changsha, 410004 China

**Keywords:** Biochemistry, Biophysics

## Abstract

Abundant nanostructures have been constantly found in various foods, like vinegar, tea, coffee, and milk. However, these structures largely remain unexplored and even been eliminated for stability reasons in food industry. Here we report the isolation, characterization, and antioxidant activities of food nanoparticles (NPs) carrying polyphenols from Chinese rice vinegar. Using a gel-chromatography-based isolation protocol, the vinegar was separated into three major fractions. They were identified as spherical NPs (P1), lollipop-like NPs (P2) and spherical microparticles (P3) with average hydrodynamic diameter of 210, 245,1643 nm, separately. The former two fractions accounted for the major parts of dry matter in the vinegar. The P1-NPs fraction was composed of proteins, carbohydrates, and a high number of polyphenols (15 wt%), demonstrated potent antioxidant activity as determined by ABTS and ORAC assays. Moreover, they effectively quenched peroxyl free radicals in peritoneal macrophages and promoted cellular growth. The P2 fraction contained majority of organic acids, esters and mineral elements of the vinegar. It demonstrated the NPs are bioactive units of the rice vinegar, inspiring the development of novel functional nanomaterials with nutraceutical and pharmaceutical applications.

## Introduction

The omnipresence of self-assembled micro/nano-particles (MNPs) within foods or traditional Chinese medicines has enjoyed a surge of attention lately^1–3^, from chicken soup, coffee, tea, fruit to herbal decoction^4^. These MNPs, composed of polar and nonpolar compositions are incidentally formed during food processing as the results of chemical reactions and physical interactions. They often serve as functional units of foods, exhibiting various biological activities. For example, MNPs from porcine bone soup can directly interact with oral and peritoneal macrophages and inhibit polarization of cells^5,6^. Many bioactive compounds have been fabricated into MNPs by various approaches, in the hope to facilitate the functions of controlled-release, targeted distribution and elevated bioavailability^7,8^. However, all these efforts are still far from application in food products, owing to the uncertainty in food safety and cost efficiency.

Besides these NPs identified from the neutral or weak acidic food matrix, an abundant amount of NPs have been found in the acidic food, vinegar^9,10^. The major steps of producing Chinese rice vinegar includes steaming of raw materials, alcohol fermentation, acetic acid fermentation, and ageing. The whole process usually lasts for more than one year, allowing the occurrence of extensive chemical reactions between carbohydrates and proteins or amino acids to form a great amount of Maillard reaction products (MRPs) and micro-/nano-scale aggregates of multiple components.

Beyond a daily used condiment, the Chinese aged rice vinegar is embraced as a traditional functional food for its health-promoting benefits, such as hypolipidemic, anti-obesity effects^11^, and microbiome regulation^12,13^. Such benefits are believed to be attributed to a great number of antioxidant components, including polyphenols, together with MRPs. The interaction of bioactive components and food or drug matrix could affect their bioactivities and functions. Yet, in what way that polyphenols in vinegar interact with human body remains unknown.

In order to improve the stability of liquid food, the industry has by all means eliminated the precipitation, in which, however, might involve the micro/nanostructures, leading to the loss of benefits of vinegar and other foods alike. Therefore, there is an urge need to isolate and characterize the micro/nanostructures in vinegar and unveil their biological activities, offering a more comprehensive insights into food nanoparticles. In this study, a separation protocol using gel-chromatography coupled with dynamic light scattering (DLS) was established for isolation of vinegar NPs. The NPs were subsequently determined for their morphological profiles, colloidal properties, compositions, antioxidant activities, and cytotoxicity.

## Result and discussions

### Gel-chromatography separation and characterization of vinegar MNPs

**Fig. 1:**
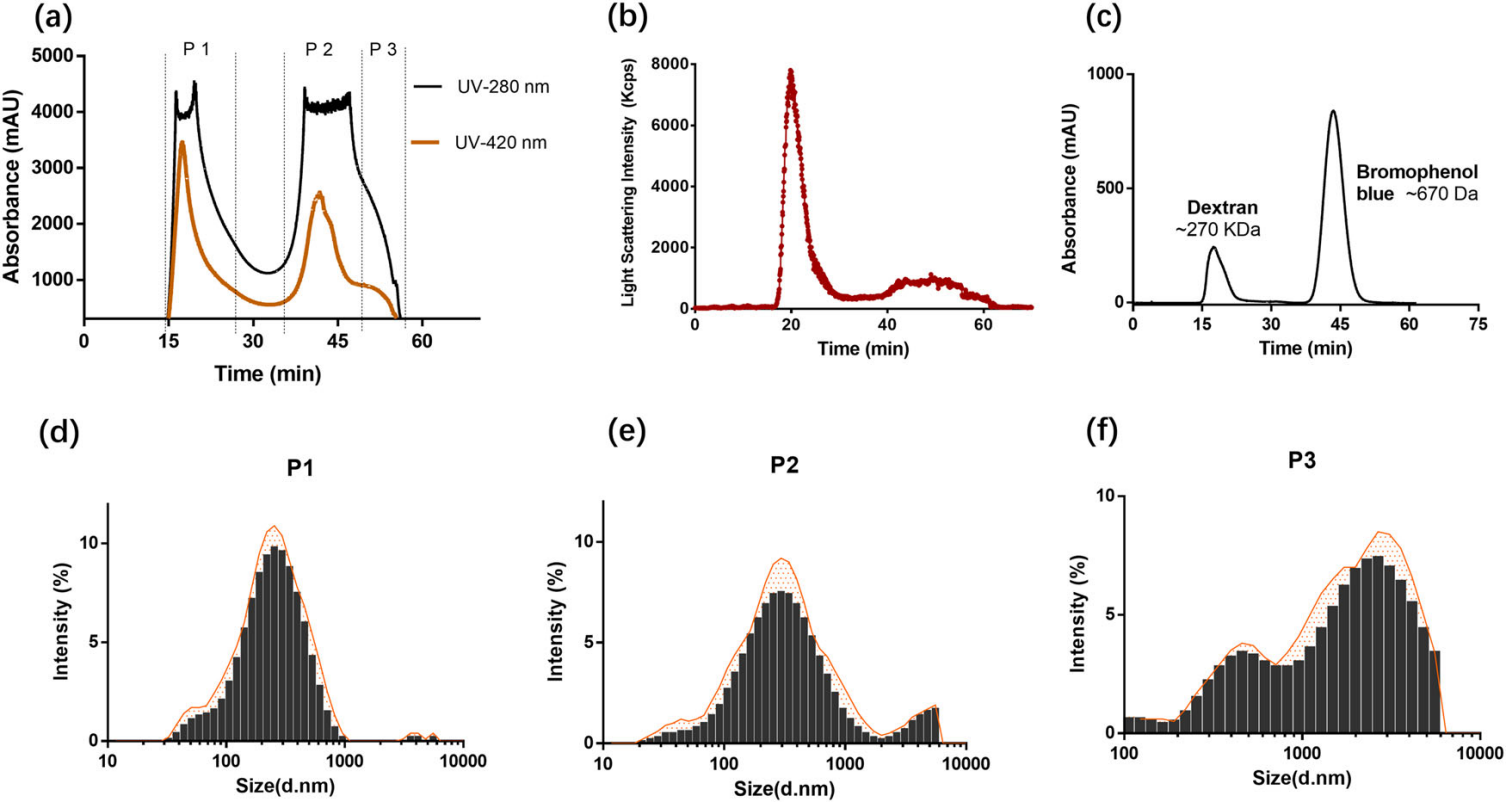
Isolation of the micro/nano-particles in vinegar with combination of size-exclusive chromatography and dynamic light scattering. **a** The isolation chromatogram. The Sepharose CL-4B column (1.0 cm × 13 cm) was used, equilibrated, and eluted with deionized water at a flow rate of 0.5 mL/min with UV absorbance at 280 nm (black line) and 420 nm (brown line). **b** Light scattering intensity of isolated micro/nanoparticles determined by the Zetasizer Nano-ZS. **c** Chromatogram obtained mixture of molecule weight standards (Dextran, Bromophenol blue). **d**–**f** Size distribution of P1-NPs, P2-NPs, and P3-MNPs.

Three fractions of Chinese aged rice vinegar were successfully separated by gel-chromatography coupled with UV absorbance (Fig. 1a) and online-DLS detector (Fig. 1b). The combined protocol has been successfully applied to the particle characterization^14,15^ and here was used to monitor the isolation of food nanoparticles from complex food matrix. In order to minimize the damage to the fractionate structures, mobile phase has to be preliminarily tested for its potential impacts on the colloidal properties of vinegar. The test results of several buffers and deionized water were determined (Supplementary Data 1). Among them, deionized water possessed the least effects on the vinegar particles while the stability might be vulnerable to the ions in buffers. Once being diluted by deionized water, the particles’ Debye screening length became larger and therefore the Coulomb repulsion between the particles would prevent the particles from aggregating and keep relatively stable. Therefore, deionized water was used as the mobile phase in the gel-chromatography separation. The first two peaks were named as P1 (elution time 15–27 min) and P2 (elution time 34–50 min), respectively, and the shoulder near P2 was named P3 (elution time 50–54 min). UV absorbance at 420 nm was used to monitor distribution of MRPs or polyphenol oxides, and UV absorbance at 280 nm were implying the presence of protein. The components in P1 scattered the laser lights most intensively among three fractions (Fig. 1b) and possessed the highest particle concentration in vinegar according to the particle number determination by Nanosight (Table 1).

**Table 1:** Colloidal properties of vinegar and fractions.

	P1	P2	P3	Vinegar
Z-average (nm)	210.4 ± 3.0	245.4 ± 6.1^**^	1643.0 ± 73.4^**^	486.7 ± 2.0^**^
PDI	0.30 ± 0.03	0.44 ± 0.02^**^	0.44 ± 0.13	0.38 ± 0.02^*^
ζ-potential (mV)	−32.20 ± 0.65	−1.37 ± 0.78 ^**^	−0.76 ± 0.02^**^	−3.40 ± 0.54^**^
Concentration (10^9^ particles/mL fraction)	14.60 ± 0.42	2.97 ± 0.63^**^	0.86 ± 0.10^**^	142.80 ± 2.07^**^

Data are presented as mean ± standard deviation (*n* = 3).

*p < 0.05 vs. P1 and **p < 0.01 vs. P1.

Right after the collection, the colloidal properties of all fractions and original vinegar were measured with DLS as shown in Table 1. The average *D*_h_ of P1-NPs, P2-NPs, and P3-NPs were 210, 245, and 1643 nm, separately. Notably, the average *D*_h_ of P1-NPs is smaller than P2-NPs and P3-NPs. However, P1-NPs, P2-NPs exhibited the same elution time with dextran (Mw = ~500 kDa) and bromophenol blue (Mw = ~670 Da), respectively (Fig. 1c), which suggested that P1-NPs should have larger size than P2-NPs and P3-NPs. One possible explanation was that the difference in the electrostatic interaction between fractions and the column packings, i.e., Sepharose led to the isolation results. The ζ-potential of P1-NPs was −32.2 mV while those of P2-NPs and P3-NPs were both near zero, as a result, P1-NPs gained larger electrostatic repulsion with column packings and then eluted before other fractions.

**Fig. 2:**
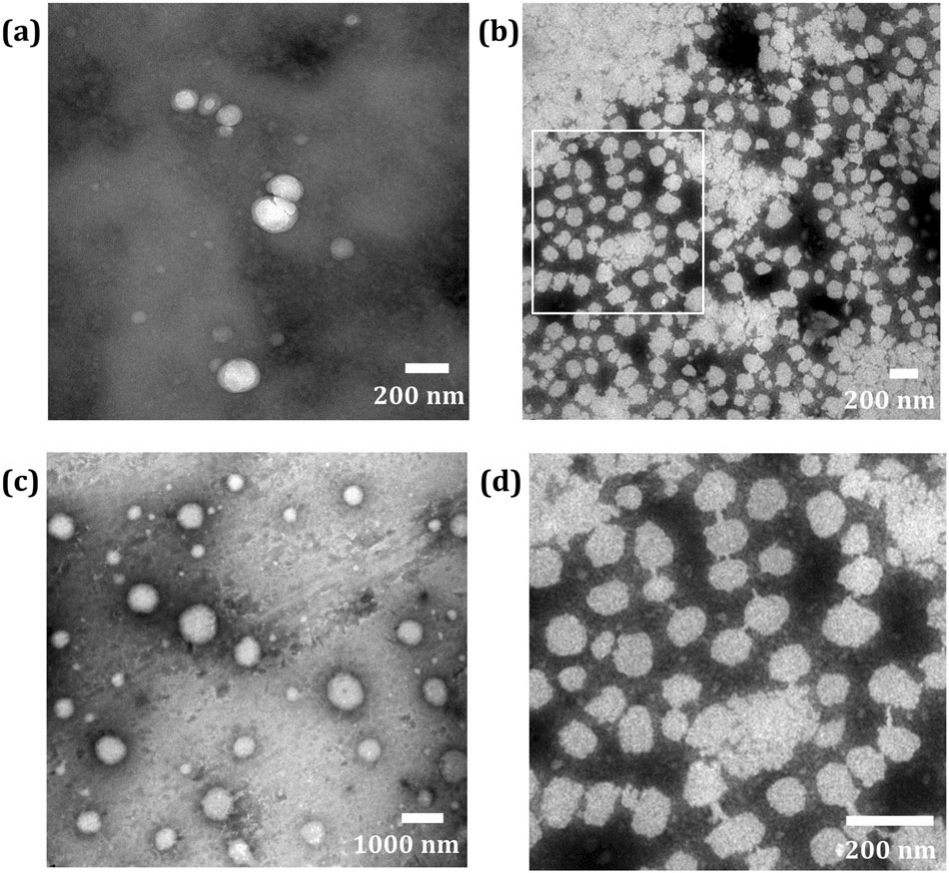
TEM micrographs of the fractions isolated from vinegar. **a** P1-NPs, original magnification ×40,000, **b** P2-NPs, original magnification ×40,000, **c** P3-MNPs, original magnification ×10,000, and **d** Selectively amplifying scope of P2-NPs.

**Fig. 3:**
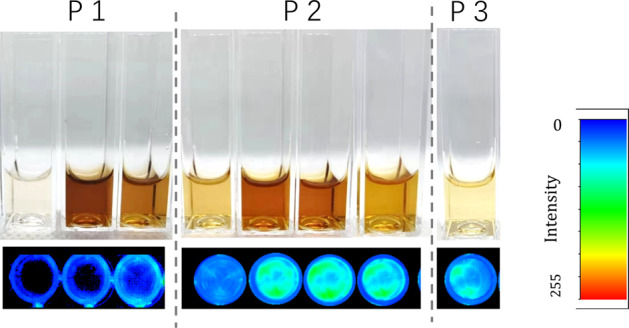
Fluorescence imaging of the isolated fractions. The images were photographed with a fluorescence stereomicroscope FluorVivo under daylight and the excitation of blue light 410–440 nm, respectively, and treated with pseudo-color.

Compared with size distribution of vinegar and other fractions, P1-NPs exhibited narrower distribution (Fig. 1d–f) and contained less microparticles. TEM observation (Fig. 2) confirmed the particle size measured by DLS and revealed some well-defined morphological details of the nano-assemblies: spherical nanoparticles in P1, lollipop-like nanoparticles in P2 and microparticles in P3. In addition, a significant number of fluorescent components were found to be distributed mainly in P2 (Fig. 3). The difference of fluorescent intensity lying in the vinegar fractions implied that at least two types of MRPs existing in vinegar. Furthermore, as fluorescent carbon nanodots with size less than 20 nm have been identified in the vinegar^9^, it is quite possible that P2 contained such nanodots, which might be derived from MRPs and could further aggregate into nanoparticles^13^.

The content of P1-NPs was about 4.5 mg per milliliter vinegar, which was over 1000 times higher than the MNPs from vinegar reported previously^10^. Low yield of these relatively small NPs (~20 nm) from the same type of vinegar may be attributed to the sample extraction using ethanol, which would cause agglomeration or decomposition of the native NPs. The chromatography-DLS separation employed here provides a less-invasive approach for isolating nanoparticles from complex food matrix.

### Compositions of vinegar NPs

The major compositions of vinegar NPs were determined and presented in Table 2. Carbohydrate and protein accounted for over 85% of P1-NPs compositions, forging the protein-carbohydrate hybrid nanostructures. In contrast, P2 was mainly composed of carbohydrates which might be low-molecular-weight, e.g., mono/oligosaccharides, organic acids, esters and a small proportion of proteins. Organic acids and esters, as the main flavor substances of vinegar, were mainly distributed in P2 and P3, but rarely in P1. The Chinese aged rice vinegar is rich in organic acids, i.e. acetic acid, lactic acid, succinic acid, oxalic acid, and aromatic acids such as sinapic acid^16^. At the same time, in the fermentation process of vinegar, the esters formed by amino acid components may be amphiphilic since they have both amino and ester bonds, resulting in the generation of specific structures such as micelles or microemulsions.

**Table 2:** Major composition of the fractions.

	P1	P2	P3
Carbohydrate (%)	31.9 ± 1.1	37.0 ± 0.4**	36.6 ± 0.9**
Protein (%)	53.1 ± 0.7	1.5 ± 0.1**	0**
Total acid (%)	0	39.1 ± 1.4**	14.0 ± 0.8**
Total ester	2.6 ± 0.6	12.3 ± 1.7**	20.3 ± 1.3**
Mineral element (%)	2.6 ± 0.4	9.1 ± 0.1**	23.8 ± 1.1**
Mass weight (mg)	4.5 ± 0.1	185.0 ± 4.3**	2.0 ± 0.2**

Data are presented as mean ± standard deviation (*n* = 3).

*p < 0.05 vs. P1 and **p < 0.01 vs. P1.

Like the organic acids and esters, the majority of minerals of vinegar was found in P2 and P3, while less than 1% of which was in P1 (Table 3). Among all the metal ions, sodium and potassium are the most dominant ones in all the three fractions, followed by magnesium, calcium, and iron. These differences may affect the particle formation and stability of the vinegar components. P1-NPs hardly contained minerals and then its ζ-potential enlarged due to the growing Debye screening length with dilution, while other fractions had high composition of minerals which might enable the particles tend to be aggregated. In this sense, P2-NPs and P3-NPs should be the precursors of precipitate in vinegar. It provides an explanation as well to the dramatical changes in the properties of vinegar caused by addition of buffers (Supplementary Data 1).

**Table 3:** Major mineral element composition of the fractions.

Mineral element (mg/L)	Detection limit	P1	P2	P3
Na	0.0015	104.20 ± 0.92	8554.67 ± 36.95**	314.13 ± 0.92**
K	0.0021	0.28 ± 0.07	5600.00 ± 84.66**	166.67 ± 1.22**
Mg	0.0009	0.24 ± 0.00	2154.67 ± 18.48**	84.53 ± 0.46**
Ca	0.0093	0	430.40 ± 2.08**	23.03 ± 0.20**
Fe	0.0007	11.16 ± 0.70	92.80 ± 1.93**	3.57 ± 0.04**
Zn	0.0002	0.51 ± 0.01	35.44 ± 1.20**	1.68 ± 0.01**
Al	0.0015	1.49 ± 0.26	16.28 ± 0.69**	0.71 ± 0.01**
Total	/	117.88 ± 1.95	16884.25 ± 145.99**	594.31 ± 2.87**

Data are presented as mean ± standard deviation (*n* = 3).

*p < 0.05 vs. P1 and **p < 0.01 vs. P1.

The size of particles in P3 fraction is not stable during the separation. It was less than 20 nm determined by online-DLS, but over 1 μm determined by static DLS. As its content in vinegar is extremely low, it was not further studied in this work.

### Bioactive compounds

Phenolic compounds are widely regarded as the main bioactives of vinegar exhibiting various health benefits, including antibacterial, antioxidant, and hypolipidemic activities. As shown in Table 4, the contents of polyphenols and flavonoids in P1 and P2 were 15.5% and 2.0%, respectively. It is rather unusual since the phenolic compounds were small molecules and not supposed to be eluted in the free form together with P1-NPs during size-exclusive separation, unless they were bound to the NPs. This binding was tough enough to resist the mechanical forces and intermolecular interactions during chromatographic separation. In this sense, phenolic compounds possibly took part in the assembly of nanostructures.

**Table 4:** Contents of flavonoids and phenolic compounds and antioxidant activities of P1 and P2.

	P1	P2
Total flavonoid (%)	6.82 ± 0.56	0.87 ± 0.02**
Total phenols (%)	8.67 ± 0.80	1.17 ± 0.08**
ABTS (mmol/g)	0.83 ± 0.11	0.29 ± 0.07**
ORAC (mmol/g)	1.19 ± 0.07	0.16 ± 0.03**

Data are presented as mean ± standard deviation (*n* = 3).

*p < 0.05 vs. P1 and **p < 0.01 vs. P1.

Among over ten kinds of phenolic compounds have been identified in the vinegar, half of which are catechins^16^. The content of phenolic compounds in P1 was 7.5 times of that in P2. Vinegar NPs here serve as nanocarriers with desirably high loading capacity for bioactive compounds compared to other delivery platforms, being expected to elevate bioaccessibility and bioavailability. As a matter of fact, the majority of existing micro/nanocarriers have a rather low loading capacities (up to 4.0 wt %^17^) while enduring complicated fabrication processes and safety risks. In addition, phenolic compounds can attach to MRPs via non-covalent bonds to reinforce their antioxidant activities^18^. Therefore, the co-existence of polyphenols and MRPs in P1 and P2 NPs may enhance antioxidant capacity of the vinegar.

Meanwhile, the polyphenols may facilitate the self-assemble of micro/nanostructures via covalent bonds reacted with MRPs^19,20^ or non-covalent bonds, e.g. π-π stacking interaction^21^ with hydrophobic moiety of proteins^22^. Moreira, et al.^23^ also reported the sugar chains, i.e., galactomannan, could polymerize with phenolic compounds in MRPs. One of the possible products of this polymerization is lollipop-like aggregates, like the NPs in P2.

### Fourier transform infrared (FT-IR) spectroscopy

**Fig. 4:**
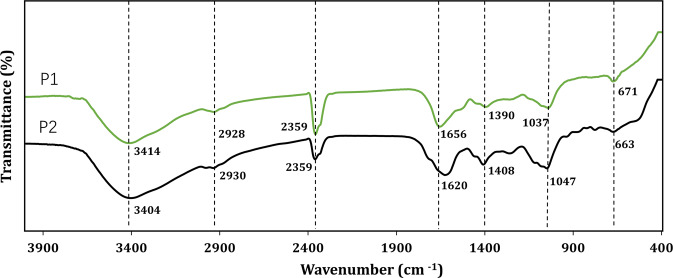
IR spectra of P1 and P2 isolated from vinegar. Main absorbance peaks were described using annotations below the spectrum.

FT-IR spectra of P1 and P2 was shown in Fig. 4. The absorbance peak around 3414 cm^−1^ is ascribed to O–H vibration and the peaks at 2930 cm^−1^ are ascribed to CH_3_ and CH_2_, respectively. Bands around at 1650, 1540 and 1400 cm^−1^ could be assigned to Amide I (C=O group), Amide II (N–H bending) and Amide III (C–N stretching), separately. One major difference between P1 and P2 fell in the range of 1620–1656 cm^−1^. The peak around 1620 cm^−1^ of P2 was attributed to –COOH vibration^9^, which is corresponded with its high content of organic acid. In contrast, P1 contains no carboxylic acids as determined in Table 2.

Bands between 1047 and 1037 cm^−1^ were caused by C–O and C–C stretching of carbohydrates, polysaccharides, or flavonoids^24^. At around at 671 cm^−1^, C–H external bending vibration of aromatic hydrocarbons^25^ was observed, validating existence of phenolic compounds in vinegar NPs.

### Antioxidant activities

To verify the antioxidant activity of P1 and P2, the ABTS cation radical reducing activity and ORAC were determined. As shown in Table 4, the activities of P1 and P2 on scavenging ABTS cation radicals were 830 ± 110 mmol trolox equivalent (TE)/g and 290 ± 70 mmol TE/g, respectively. Their ORAC activities were 1190 ± 70 mmol TE/g and 160 ± 30 mmol TE/g, respectively. It elucidates that P1 and P2 are contributing to the antioxidant capacity of vinegar. The antioxidant activity of P1 was a few times higher than that of P2, echoing the 7.5 times higher content of polyphenols in P1. The difference in ABTS and ORAC might be attributed to the different capacity of vinegar components on scavenging ABTS cation radicals and peroxyl radicals^26^. Besides phenolic compounds, oligosaccharides and organic acids may reduce ABTS radicals, too. Furthermore, the binding of phenolic compounds to the MNPs may improve the former’s physicochemical stability and bioavailability.

### CAA

**Fig. 5:**
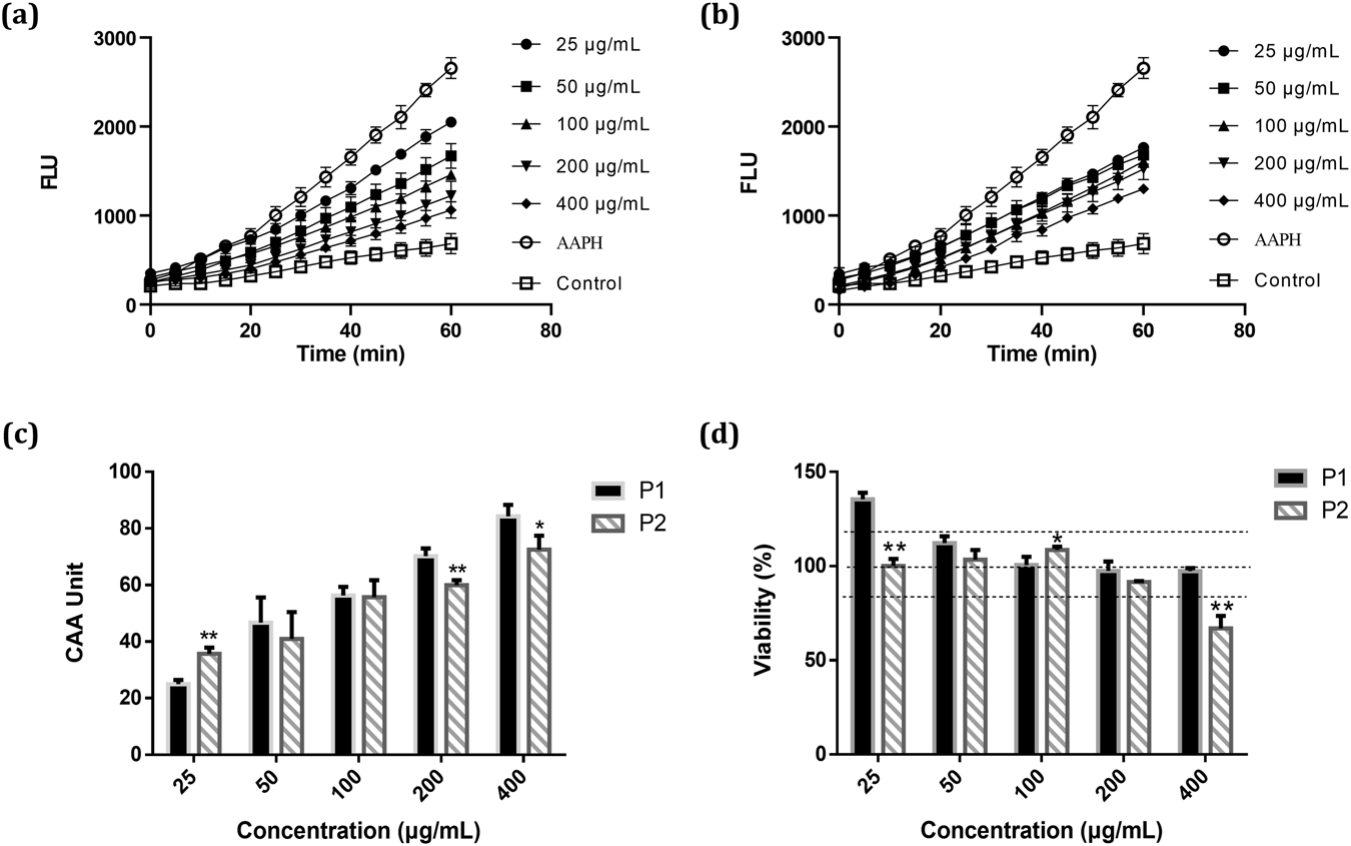
The effects of P1 and P2 on the cellular redox status and viability of peritoneal macrophages. **a** Kinetic curves of AAPH-induced DCF fluorescence and the inhibition of oxidation by P1 or **b** P2 on peritoneal macrophages from mice. **c** Cellular antioxidant activity of P1 or P2 with different concentration on peritoneal macrophages. **d** Viability of peritoneal macrophages incubated with P1 or P2 for 24 h and measured by MTT assay (*n* = 3). Data are presented as mean±standard deviation. Statistics: *p < 0.05 vs. P1 and **p < 0.01 vs. P1, *t*-test.

In CAA assay, DCFH-DA derived fluorescence is selectively reporting the level of intracellular reactive oxygen species (ROS). As shown in Fig. 5a, b, the AAPH-induced intracellular ROS of macrophages were significantly decreased by P1 and P2, respectively, indicating potent cellular antioxidant activities in both P1 and P2. As shown in Fig. 5c, both P1 and P2 showed dose-dependent antioxidant activities on macrophages, e.g. CAA value over 60 unites at 200 μg/mL. The CAA of P1 was higher than that of P2, though marginally, implying the important contribution of NPs to bioactivities of the vinegar. Despite the much lower content of polyphenol, P2 exhibited similar cellular antioxidant activities with P1. For the CAA, the free radical scavenging capacity and initial concentration of bioactives did not waltz alone. The cellular uptake, distribution and metabolism of bioactive components, often altered by ζ-potential and micro-/nano-structure of NPs, all affect their final biological impacts^27,28^.

Macrophages play essential roles in the innate immune response and tissue homeostasis^29^. Food-derived NPs have demonstrated regulatory effects on immune cells including macrophages. Colloidal particles in bone soup were quickly engulfed by oral and peritoneal macrophages and maintain normal mitochondrial metabolism^5^. NPs from grape could protect mice against dextran sulfate sodium induced colitis by modulating stem cells and macrophages^30^. The modulation of macrophage proliferation and intracellular ROS by vinegar NPs is indicating their potential immune-regulatory effects.

### Cytotoxicity

As shown in Fig. 5d, the influences of P1 and P2 on viability of murine peritoneal macrophages were determined with MTT assay. P1-NPs showed no cytotoxicity at all the assayed concentrations, as high as 400 μg/mL while promoted cell proliferation by 35% at the lowest concentration of 25 μg/mL. While the active principles in P1 NPs responsible for their cell proliferation promoting activity are awaiting to be identified, one may expect the rich content of polyphenols in these NPs could be one of the underlying reasons. In contrast, P2 was nontoxic to the cells at the concentration range of 25–200 μg/mL, while inhibited cell proliferation by over 30% at 400 μg/mL. The high content of organic acids in P2 should be responsible for its cytotoxicity at the high concentration. The tested concentration (25–400 μg/mL) of P1 and P2 was roughly equivalent to 6–90 μL and 0.1–2.2 μL of vinegar, respectively. It suggested that the particles in vinegar may directly affect the mucosal cells in the upper part of alimentary tract, e.g., macrophages in the mouth, even at a very low dose.

The nanocarriers often provide protection, targeted delivery, elevated bioavailability, and bioactivities to their cargo compositions^31^. Moreover, the bioatives in vinegar are highly concentrated on the nanoparticles rather than dispersed evenly in the aqueous phase. Taking polyphenols in vinegar as example, they were condensed in NPs with a concentration over 14,000 times higher than that of overall vinegar dispersion, as the NPs only occupy 1/14000 of dispersion’s total volume. The effect of concentrated bioactives bound to NPs validates the new possibility that NPs at a low dose can exert considerable bio-functions and provides with a new interaction mechanism between food NPs within human bodies. As a price, the bioactive payloads may be unintentionally discarded during the production and consumption as the vinegar NPs gradually agglomerated and precipitated, possibly triggered by the low surface potential of particles in the acidic solution.

Although the vinegar NPs have shown significant impact on peritoneal macrophages in vitro, the real situation when they encounter the mucosal cells in digestive tract, together with other components of food and mucosal fluids, is a fascinating and meaningful subject for the future studies.

**Fig. 6:**
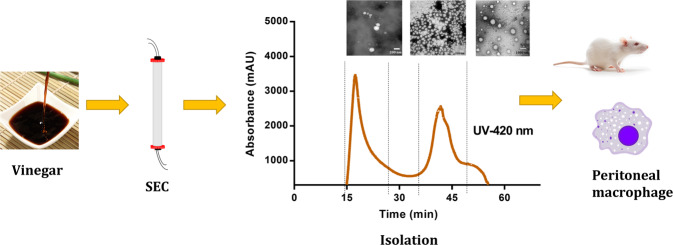
Graphical illustration of the isolation, characterization, and antioxidant activities detection for vinegar nanoparticles. Briefly, the vinegar was centrifuged at 5,000 g for 15 min to remove the precipitates. The supernatant was then injected into the gel-chromatography column (1.0 cm × 13.0 cm) coupled with DLS and UV detection. Subsequently, three fractions were observed in the chromatogram. The colloidal properties, chemical composition, and representative TEM image of fractions were determined. At last, the effects of the major fractions on the cellular redox status and viability of peritoneal macrophages from SD mice were explored.

In conclusion, spherical nanoparticles, lollipop-like nanoparticles and microparticles were successively isolated from vinegar by DLS coupled gel-chromatography. Vinegar NPs were nano-vehicles with high loading capacity for hydrophobic and hydrophilic antioxidants, exerting potent oxygen-free radical scavenging capacity and cellular antioxidant activity on mucosal macrophages (Fig. 6). Once these NPs are removed, the function and stability of original foods would inevitably be affected. Beyond single molecules, the food nanoparticles acquire a revisit to the quality and function evaluation of vinegar, expanding our understanding of this traditional fermented food and other liquid foods such as tea, coffee, and fruit juice. Moreover, utilizing the food nanoparticles as functional carriers, a novel approach is emerging for development of effective, safe, and sustainable nano-scale food ingredients.

## Methods

### Materials

Vinegar was provided by Jiangsu Hengshun Vinegar-Industry Co., Ltd (Jiangshu, China), which is one of the representative rice vinegars in China. Deionized water was prepared by a Millipore Milli-Q water system (Millipore, Bedford, MA, USA). Blue Dextran (MW: ~500 kDa), and Bromophenol blue (MW: ~670 Da), 2,2-azobis(2-amidinopropane) hydrochloride (AAPH), 3-(4,5-dimethyl-2-thiazolyl)-2,5-diphenyl-2-H-tetrazolium bromide (MTT) and DCFH-DA ( ≥ 97% purity) were purchased from Sigma-Aldrich Co., Ltd. (Shanghai, China). ABTS kit was purchased from Beyotime Biotechnology Co., Ltd (Shanghai, China). DMEM, FBS, streptomycin, PBS and penicillin were purchased from Thermo Fisher Scientific (China) Co., Ltd (Shanghai, China). All the chemicals and solvents, unless otherwise noted, used were of AR grade, and Sepharose CL-4B column (60–2 × 10^4^ kDa) were purchased from Sinopharm Chemical Reagent Co., Ltd (Shanghai, China).

### Isolation of vinegar NPs by gel-chromatography coupled DLS

The vinegar was centrifuged at 5000 × *g* for 15 min to remove the precipitates. The supernatant was collected and stored at −4 °C until analyzed. To minimize the damage to the fractionate structures, mobile phase was preliminarily tested for its potential impacts on the colloidal properties of vinegar. One milliliter of vinegar was diluted ten times with different buffers, separately, and the impacts of buffers on the vinegar stability then were determined by DLS (Zatasizer Nano-ZS, Malven instruments Ltd, UK). One milliliter vinegar was subjected to a pre-equilibrated *gel-chromatography* column (Sepharose CL-4B, 1.0 cm × 13.0 cm) equipped with a UPLC (Chromatography System, BIO-RAD, USA) system at flow rate 0.5 mL/min, using deionized water as mobile phase and UV monitor at 280 and 420 nm, respectively, and coupled with a DLS instrument (Zetasizer Nano-ZS, Malven instruments Ltd, UK) equipped with a flow-cell. Elutes were collected by an automatic fraction collector at 2 mL/tube (NGC Fraction Collector, BIO-RAD, USA). As shown in Fig. 1, the fractions with strong UV absorbance were collected and labeled as below: P1 (elution time 15–27 min), P2 (elution time 34–50 min), and P3 (elution time 50 to 54 min). The fractions were lyophilized by a lyophilizer (VCP63MV, Martin Christ GmbH, Osterode, Germany) and stored at −30 °C.

### Colloid properties and morphology

The hydrodynamic diameter, polymer dispersity index (PDI), and ζ-potential of the three fractions and vinegar were characterized by DLS (Zatasizer Nano-ZS, Malven instruments Ltd, UK). The particle numbers and size distribution of these samples were determined by Nanosight LM10 (Malven instruments Ltd, UK). One milliliter of the supernatant, without dilution, was gently injected to the cuvette for measurement at 25 °C. Viscosity of 0.8872 and refractive index of 1.330 were used in the measurement and analysis.

TEM observation of fractions was performed with JEOL JEM-1230 (TEM, Japan). The dispersion was transferred to the 230 mesh copper grid, stained with phosphotungstic acid, and then observed under the TEM at 80 kV.

### Fluorescence imaging

Three fractions were photographed with a fluorescence stereomicroscope FluorVivo (INDEC BioSystems, USA) under daylight and the excitation of blue light 410–440 nm. The fluorescent images were treated with pseudo-color according to the fluorescent intensity.

### Major composition analysis

Protein concentration was determined by Coomassie Brilliant Blue assay^32^. Briefly, 0.1 mL sample or protein standard of different concentrations (0–0.8 mg/mL) and five milliliters of protein reagent (0.01% Coomassie Brilliant Blue G-250, 4.7% ethanol, and 8.5% phosphoric acid) were added to the test tube and the contents mixed by inversion. The absorbance at 595 nm was measured in the 3 mL cuvette with ultraviolet spectrophotometer (UV-5100, HITACHI, Japan).

Carbohydrate concentration was determined by anthrone-sulfuric acid assay^33^. Briefly, 0.5 mL sample or glucose standard of different concentrations (0–80 μg/mL) were added to 5 mL of anthrone reagent (0.75% anthrone and 84% sulfuric acid). The samples were shaken with a Vortex mixer (Thermofisher, USA) to ensure complete uniformity of the dispersion and heated for exactly 10 min in a boiling-water bath. They were cooled in an ice-water bath and measured the absorbance at 590 nm.

The total acid and ester content was conducted by titration method (AOAC, 1984)^34^. Briefly, 50.0 mL sample with 2 drops of phenolphthalein indicator solution was pipetted into a 250 mL Erlenmeyer flask. To neutralize the acid the sample, 0.1 mol/L sodium hydroxide standard solution was slowly added until the color became reddish. The recorded consumption of sodium hydroxide solution in milliliters then convert the total acid content. The esters in the samples were saponified with abundant and 0.1 mol/L NaOH solution, and then the residual NaOH was titrated with 0.1 mol/L H_2_SO_4_ solution. The content of esters could be calculated by the quantitative relationship with actual consumed H_2_SO_4_ in the chemical reaction.

The mineral composition was evaluated by inductively coupled plasma mass spectrometry (Thermo CAPQ ICP-MS, Thermo Electron, Waltham, MA, USA). Briefly, 1.0 mL of hydrogen peroxide and 3.0 mL of nitric acid were added to 0.5 mL of sample in closed microwave digestion tank. Then the digestion was according to the following procedure that microwave power was 1500 W, temperature programming from 0 to 120 °C within 5 min (held for 5 min), continued temperature programming to 200 °C within 5 min (held for 30 min). A multi-element stock standard solution containing all the analytical mineral elements was used to prepare a standard curve. The digested samples were dissolved in deionized water and analyzed using ICP-MS.

### Bioactive compounds analysis

The total phenols contents in P1 and P2 were determined by Folin–Ciocalteu method, and the total flavonoid contents (TFC) in P1 and P2 were determined by AlCl_3_ method^16^. Briefly, samples were diluted ten times with deionized water, and 0.2 mL of diluted sample was mixed with 0.8 mL Folin–Ciocalteu reagent. After 5 min, 1.5 mL 10% Na_2_CO_3_ (w/v) solution was mixed and then deionized water was added to obtain the final volume of 10 mL. The mixture was measured at 765 nm after 120 min in the dark with Gallic acid used as a reference. Besides, every sample was neutralized with 2% NaOH solution and diluted 10 times with deionized water. Two milliliters of diluted sample, 8 mL of distilled water and 1 mL of 5% NaNO_2_ solution were mixed. After 6 min, 1 mL of 5% Al (NO_3_)_3_ solution was added and stood for 6 min. Finally, 4 mL of 20% NaOH solution was added and made up to 25 mL with deionized water. After 15 min, the absorbance was measured at 510 nm with Rutin used as a reference. All the data was recorded in a UV–visible Spectrophotometer (U-5100, Hitachi, Japan).

### Fourier transform infrared spectrometer

A Fourier transform infrared spectrometer (FT-IR-8400, Shimadzu, Kyoto, Japan) was used to record Fourier transform infrared spectra of P1 and P2 with an accumulation of 32 scans and a resolution of 1 cm^−1^.

### Extracellular antioxidant activity

#### ABTS assay

The antioxidant activities of P1 and P2 were measured by ABTS assay kit (Beyotime) according to the manufacturer’s protocols^34^. The absorbance at 734 nm was measured with a FlexStation 3 Plate Reader (Molecular Devices, USA). The data were expressed as TE/g of samples. The equation is shown as below:

$${{{\mathrm{ABTS}}}}\,{{{\mathrm{value}}}} = {{{\mathrm{Trolox}}}}\,{{{\mathrm{Equivalent/Dry}}}}\,{{{\mathrm{weight}}}}\,{{{\mathrm{of}}}}\,{{{\mathrm{sample}}}}\,\left( {{{{\mathrm{TE}}}}\,{{{\mathrm{mmol/g}}}}} \right)$$

#### Oxygen radical absorbance capacity (ORAC) assay

The antioxidant activities of P1 and P2 were measured by ORAC assay^35^. The fluorescence intensity was recorded every 2 for 120 min at Ex485 nm and Em520 nm, respectively (FlexStation 3 Plate Reader, Molecular Devices, USA). The data were expressed as TE/g of samples according to the equation as below:

$${{{\mathrm{ORAC}}}}\,{{{\mathrm{value}}}} = {{{\mathrm{Trolox}}}}\,{{{\mathrm{Equivalent/Dry}}}}\,{{{\mathrm{weight}}}}\,{{{\mathrm{of}}}}\,{{{\mathrm{sample}}}}\,\left( {{{{\mathrm{TE}}}}\,{{{\mathrm{mmol/g}}}}} \right)$$

### Preparation of murine peritoneal macrophages

Murine peritoneal macrophages were prepared by peritoneal lavage in SD mice according to a previously reported method^6^. Briefly, mice were euthanized with rapid cervical dislocation and sacrificed, and 10 mL DMEM was injected into the intraperitoneal cavity. The abdomen was gently massaged for 10 min, followed by 15 min of rest. Sterilized scissors and forceps were used to cut the outer skin of the peritoneum and the inner skin lining the peritoneal cavity was exposed. The injected medium was then aspirated. Cells were washed with DMEM medium (15% FBS, penicillin 100 U/mL, streptomycin 100 μg/mL) by centrifuge for 5 min at 400 × *g* each time. The washed cells were re-suspended in DMEM medium at room temperature and then seeded in 96-well plates (5 × 10^4^ cells per well) and cultured at 37 °C with 5% CO_2_. The animal experiment mentioned here was inspected and approved by the Animal Care & Welfare Committee of Zhejiang Academy of Medical Sciences, China (No. 2019R07001).

### Cellular antioxidant activity (CAA) assay

The CAA assay was carried out according to a previously reported method^36^ with slight modifications. Briefly, peritoneal macrophages (1.0 × 10^5^ cell/well) were seeded in 96- well black and incubated at 37 °C for 24 h. The DMEM medium (15% FBS, penicillin 100 U/mL, streptomycin 100 μg/mL) was removed and the cells were washed with HBSS (Hank’s balanced salt solution) to remove any non-adherent and dead cells. All subsequent samples (lyophilized fractions) and reagents were dissolved in DMEM medium (penicillin 100 U/mL, streptomycin 100 μg/mL) for the next performances. Sample solution (100 μL) and DCFH-DA (50 μM, 100 μL) were added to each well in triplicates. After 1-h incubation at 37 °C, cells were immediately washed by HBSS. Then 100 μL of AAPH (600 μM) was added to the cells and the Real-time fluorescence was immediately determined with a plate reader (FlexStation 3, Molecular Devices, USA) every five minutes for 1 h at Ex485 nm and Em538 nm. The cells in ‘positive control’ were incubated with DCFH-DA and AAPH at the absence of antioxidant. The cells in ‘blank control’ were incubated only with DCFH-DA. The CAA unit was calculated according to the equation shown as below:

$${{{\mathrm{CAA}}}}\,{{{\mathrm{unit}}}} = \left( {1 - {{{\mathrm{AUC}}}}_{{{{\mathrm{sample}}}}}{{{\mathrm{/AUC}}}}_{{{{\mathrm{control}}}}}} \right) \times 100\%$$

### Cytotoxicity

Cytotoxicity of P1 and P2 was determined by MTT assay^37^. The original cell culture in the 96-well culture plate was removed and cells were washed with HBSS. P1 and P2 were dissolved in DMEM respectively and diluted to different concentrations (25–400 μg/mL) in the plate for 24 h. Here, 400 μg lyophilized P1 or P2 was equal to 90 μL or 2.2 μL original vinegar, respectively. Next, cells were incubated with 20 μL of MTT reagent (0.5 mg/mL) at 37 °C for 4 h, and then washed twice with 1× phosphate-buffered saline. DMSO (150 μL) was added and the plate was placed on a horizontal shaker for 10 min. The absorbance of each sample was recorded at 570 nm. Each sample was tested in triplicates.

### Statistical analysis

Data presented were the mean value and standard deviation obtained from three samples. Significant differences were determined by *t*-test using Microsoft Office Excel. Significance was determined at *p* < 0.05.

## Supplementary information

The colloidal properties of vinegar and diluted vinegars by different buffer.

## Data Availability

The authors declare that the data supporting the findings of this study are available within the article.
