# Supplementary material for: Food nanoparticles from rice vinegar: isolation, characterization, and antioxidant activities
Source: NPJ Sci Food. 2022 Jan 11;6:1. doi: 10.1038/s41538-021-00118-y (PMC8752661; doi:10.1038/s41538-021-00118-y)
Supplement: Supplementary file 1 — The colloidal properties of vinegar and diluted vinegars by different buffer. [file 41538_2021_118_MOESM1_ESM.pdf]

Supplemental Information

Supplementary Table 1. The colloidal properties of vinegar and diluted vinegars by different buffers

Diluted vinegar	Size（nm）	PDI
Vinegar	486.7±2.0	0.38±0.02
Deionized water	431.1±4.1	0.51±0.04
pH 3.6，0.01M Sodium acetate -Acetic acid buffer	547.9±14.2	0.49±0.01
pH 3.6，0.02M Sodium acetate -Acetic acid buffer	981.8±89.8	0.52±0.02
pH 5，0.01M Sodium acetate -Acetic acid buffer	782.6±9.2	0.45±0.10
pH 7，0.01M Phosphate buffer	316.5±5.7	0.48±0.03
